# Bioequivalence study of three ibuprofen formulations after single dose administration in healthy volunteers

**DOI:** 10.1186/1471-2210-8-18

**Published:** 2008-10-29

**Authors:** Peter Bramlage, Adrian Goldis

**Affiliations:** 1 Institute for Clinical Pharmacology, Medical Faculty Carl Gustav Carus, Technical University Dresden, Fiedlerstrasse 27, 01307 Dresden, Germany; 2 IFE Human Pharmacology SRL, Str. Cornelia Salceanu Nr. 5, 300561 Timisoara, Romania

## Abstract

**Background:**

This phase I study was designed to determine the bioavailability and bioequivalence of 400 mg Eudorlin^® ^extra* (Ibuprofen) in comparison to two reference formulations (400 mg Nurofen^® ^forte and 400 mg Migränin^® ^after single dose administration under fasting conditions in healthy subjects. Therefore the design of a randomized, open label, multiple sequence cross-over study with a wash-out period of 7–10 days was used.

**Results:**

AUC_0-t(last) _and AUC_0-∞ _(90%CI) were within the 80 to 125% interval required for bioequivalence as stipulated in the current regulations of the EMEA. C_max _(90%CI) was within the EMEA acceptance range of 75 to 133%. Detailed analyses showed that C_max _of Eudorlin^® ^extra was higher than that of Nurofen^® ^forte (36.62 vs. 32.92 μg/ml; p = 0.0014) and that of Migränin^® ^(35.94 vs. 30.87 μg/ml; p < 0.0001). The time to maximum plasma concentration (t_max_) was shorter with Eudorlin^® ^extra than with Nurofen forte (1.14 vs. 1.82 h; p < 0.0001) and Migränin (1.13 vs. 1.78 h; p = 0.0031). Only 1 patient experienced an adverse with possible relation to the study drug taking Migränin^®^.

**Conclusion:**

It is concluded that Eudorlin^® ^extra is bioequivalent to the two reference preparations Nurofen^® ^forte and Migränin^® ^for both, the extent and the rate of absorption, after single dose administration in healthy volunteers according to the guidance of the EMEA. Within this frame, peak plasma concentrations are however reached earlier and peaks are higher compared to the reference products.

* Eudorlin^® ^extra may have different brand names in different countries

## Background

Ibuprofen was developed during the 1950s and 1960s as a 'super aspirin' for the treatment of rheumatoid arthritis which was as effective as current alternatives but safer. First synthesised in December 1961 ibuprofen was found to have a short elimination half-life and exceptional gastrointestinal tolerability [1]. Ibuprofen is mostly used today in the management of mild to moderate pain and inflammation in conditions such as dysmenorrhoea, headache including migraine, postoperative pain, dental pain, musculoskeletal and joint disorders such as ankylosing spondylitis, osteoarthritis, and rheumatoid arthritis. It is also used to reduce fever. The usual oral dose in adults is 400 to 800 mg daily for analgesia and up to 1600 to 2400 mg for its anti-inflammatory action [2].

Ibuprofen is absorbed from the gastro-intestinal tract and peak plasma concentrations are reached within about 1 to 2 hours after ingestion. Bioavailability is ≥ 80%. 99% of ibuprofen is bound to plasma proteins, 90% is transformed to 2 inactive metabolites and it has a plasma half-life of about 2 ± 0.5 hours. It is rapidly excreted in the urine mainly as metabolites and their conjugates. About 1% is excreted in urine as unchanged ibuprofen [2].

The European Agency for the Evaluation of Medicinal Products (EMEA) requires generic products that enter the marketplace to show bioequivalence to assess the possibility of alternative use between the reference product and an essentially similar medicinal product [3]. This is done assuming that in the same subject an essentially similar plasma concentration time course will result in essentially similar concentrations at the site of action and thus in an essentially similar effect. Medicinal products authorized and marketed on the basis of a full dossier i.e. including chemical, biological, pharmaceutical, pharmacological-toxicological and clinical data are used as the reference product.

The present study was designed to investigate the bioavailability and the bioequivalence of 400 mg Eudorlin^® ^extra in comparison to two reference products – Migränin^® ^and Nurofen^® ^forte after single oral administration (fasting conditions) in healthy subjects following the EMEA guidance [3]. Two reference products were used since different reference formulations were required in different countries. For this purpose the rate and extent of absorption of ibuprofen under fasting conditions after administration of one tablet of the test product (Eudorlin^® ^extra) and one tablet of each of the reference products were compared in a three sequence cross-over design.

## Results

### Demography and baseline characteristics

Between February 3^rd ^and March 8^th ^2007 86 subjects were screened for eligibility of whom 60 were enrolled, randomized and available for pharmacokinetic and safety analyses. 41 of these were male, 19 female. There were three protocol violations (Per-protocol population n = 57). For the first patient the labelling of two blood samples was missing, the other two patients missed at least one visit with missing information for that particular time point. Table 1 displays the patient characteristics of the pharmacokinetic population by treatment sequence. A refers to reference product Nurofen^® ^forte, B to reference product Migränin^® ^and E to test product Eudorlin^® ^extra.

**Table 1:** Demographic data of subjects

**Parameter**	**Sequence**	**N**	**Mean**	**SD**	**Median**	**Range**
Age (years)	BE	20	23.9	3.65	23.5	20; 35
	EA	20	25.1	5.01	24.0	20; 39
	AEB	20	27.8	6.57	26.5	20; 45

Weight (kg)	BE	20	68.3	10.74	69.5	50; 90
	EA	20	67.7	9.54	68.5	49; 82
	AEB	20	67.9	10.48	68.0	52; 87

BMI (kg/m^2^) *	BE	20	22.8	2.40	23.0	19; 27
	EA	20	22.9	2.29	23.0	19; 27
	AEB	20	22.2	2.67	22.0	19; 27

						

	**Sequence**	**N**	**N (♀)**	**% (♀)**	**N (♂)**	**% (♂)**

Female gender (%)	BE	20	7	35.0	13	65.0
	EA	20	6	30.0	14	70.0
	AEB	20	6	30.0	14	70.0

Demography and other baseline characteristics by treatment sequence (Pharmacokinetic population; N = 60). "A" refers to Nurofen^® ^forte, "B" refers to Migränin^® ^and "E" refers to Eudorlin^® ^extra. * calculated from measured height (m) and weight (kg).

### Dissolution testing

**Figure 1:**
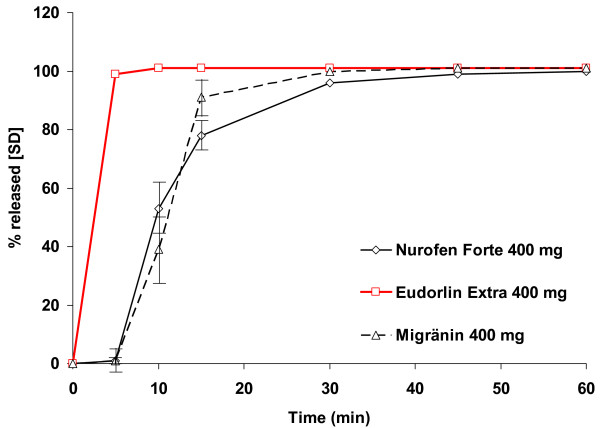
**Results of dissolution testing**. Dissolution testing of 400 mg tablets Eudorlin^® ^Extra (Test formulation, in red) versus 400 mg Nurofen^® ^Forte (Reference formulation 1, black) and 400 mg Migränin^® ^(Reference formulation 2, broken black).

Results of dissolution testing are displayed in Figure 1. Testing was performed for the three products using a total of 504 samples for different time points. All samples resulted in values between 0 and a maximum of 104%. Dissolution was particularly fast with Eudorlin^® ^extra resulting in 99% released ibuprofen within 5 minutes. About the same dissolution was observed after 45 minutes with Nurofen^® ^forte (99%) and 30 minutes using Migränin^® ^(100%).

### Pharmacokinetic

Table 2 displays the pharmacokinetic parameters of the pharmacokinetic (PK) population (n = 60). Table 3 illustrates the statistical analysis. AUC_0-t(last) _and AUC_0-∞ _was similar for the comparison of Eudorlin^® ^extra with Nurofen^® ^forte and Eudorlin^® ^extra with Migränin^®^. All estimates of the ratios for the parameters AUC_0-t(last) _and AUC_0-∞ _were near to 1.00 and the 90%-confidence intervals for all comparisons were within the acceptance range of 0.80 – 1.25.

**Table 2:** Pharmacokinetic parameters

**Parameter analyzed**	**Sequence**	**Treatment**	**Mean**	**SD**	**CV (%)**	**Geometric mean**
AUC_0-t(last) _(μg/ml*h)	AE/EA	Nurofen^®^	117.00	31.94	27.3	113.4
		Eudorlin^®^	119.79	29.04	24.2	116.6
	EB/BE	Migränin^®^	115.21	21.83	19.0	113.1
		Eudorlin^®^	115.84	22.73	19.6	113.7

AUC_0-∞ _(μg/ml*h)	AE/EA	Nurofen^®^	117.38	32.01	27.3	113.8
		Eudorlin^®^	120.15	29.07	24.2	117.0
	EB/BE	Migränin^®^	115.57	21.84	18.9	113.5
		Eudorlin^®^	116.22	22.73	19.6	114.1

C_max _(μg/ml)	AE/EA	Nurofen^®^	32.92	8.29	25.2	31.83
		Eudorlin^®^	36.62	6.16	16.8	36.11
	EB/BE	Migränin^®^	30.87	6.31	20.4	30.25
		Eudorlin^®^	35.94	6.30	17.5	35.41

t_1/2 _(h)	AE/EA	Nurofen^®^	2.52	0.44	17.6	2.495
		Eudorlin^®^	2.55	0.42	16.6	2.522
	EB/BE	Migränin^®^	2.50	0.33	13.1	2.478
		Eudorlin^®^	2.66	0.79	29.8	2.583

						

			**Mean**	**SD**	**CV**	**Median**

t_max _(h)	AE/EA	Nurofen^®^	1.82	1.05	57.9	1.50
		Eudorlin^®^	1.14	0.67	59.1	1.00
	EB/BE	Migränin^®^	1.78	0.97	54.5	1.75
		Eudorlin^®^	1.13	0.80	71.1	0.75

Pharmacokinetic data by treatment pair and treatment (Pharmacokinetic population; N = 60). "A" refers to Nurofen^® ^forte, "B" refers to Migränin^® ^and "E" refers to Eudorlin^® ^extra. SD: standard deviation, CV: coefficient of variation.

**Table 3:** Statistical analysis for AUC_0-t(last)_, AUC_0-∞ _and C_max_

**Parameter**	**Ratio**	**Estimate**	**90%CI**
AUC_0-t(last)_	E/A	1.0241	0.9959; 1.0531
	E/B	1.0058	0.9700; 1.0430
AUC_0-∞_	E/A	1.0240	0.9958; 1.0529
	E/B	1.0060	0.9704; 1.0430
C_max_	E/A	1.1084	1.0542; 1.1852
	E/B	1.1732	1.1058; 1.2447

			

**Parameter**	**Difference**	**Estimate**	**90%CI**

t_max_	E – A	-0.6250	-0.8750; -0.3750
	E – B	-0.6875	-1.000; -0.3750

Statistical analysis by treatment pair (parametric analysis, pharmacokinetic population; N = 60). "A" refers to Nurofen^® ^forte, "B" refers to Migränin^® ^and "E" refers to Eudorlin^® ^extra. CI: confidence interval.

The mean C_max _of ibuprofen differed after Eudorlin^® ^extra compared to Nurofen^® ^forte (36.62 vs. 32.92 μg/ml; p = 0.0014) and after Eudorlin^® ^extra compared to Migränin^® ^(35.94 vs. 30.87 μg/ml; p < 0.0001). The 90%-confidence intervals for both comparisons were however within the widened acceptance range of 0.75 – 1.33 (pre-defined), but also within the standard acceptance range of 0.80 – 1.25.

The t_max _varied for Eudorlin^® ^extra between 0.25 to 3.50 hours and after treatment with Nurofen^® ^forte or Migränin^® ^between 0.50 and 5.00 hours (Table 2 displays the mean values ± standard deviation). The difference in t_max _of Eudorlin^® ^extra and Nurofen^® ^was statistically significant (1.14 vs. 1.82 h; p < 0.0001) as was the difference between Eudorlin^® ^extra and Migränin^® ^(1.13 vs. 1.78; p = 0.0031).

As an additional variable the elimination half life t_1/2 _was calculated. The mean elimination half life t_1/2 _values were similar for all treatments with 2.52 hours after Nurofen^® ^forte, 2.50 hours after Migränin^® ^and slightly higher after Eudorlin^® ^extra with 2.55 or 2.66 hours compared to Migränin^® ^or Nurofen^® ^forte respectively.

**Figure 2:**
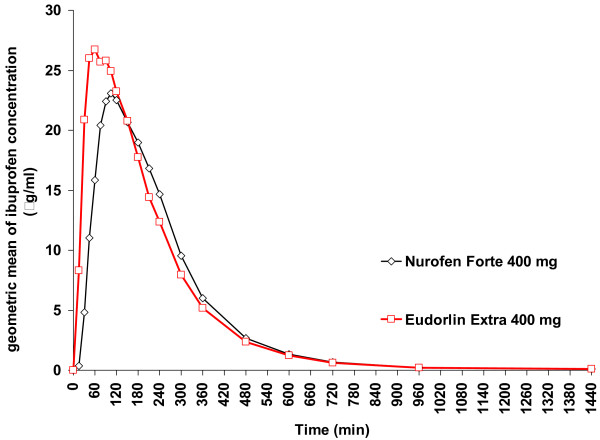
**Plasma concentration time curve Eudorlin^® ^vs. Nurofen^®^**. Geometric mean time curve for plasma concentration of ibuprofen; cross-over Eudorlin^® ^extra – Nurofen^® ^forte (Pharmacokinetic population; N = 60).

**Figure 3:**
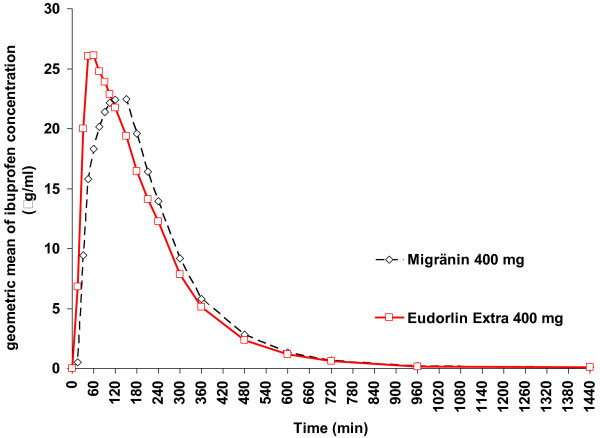
**Plasma concentration time curve Eudorlin^® ^vs. Migränin^®^**. Geometric mean time curve for plasma concentration of ibuprofen; cross-over Eudorlin^® ^extra – Migränin^® ^(Pharmacokinetic population; N = 60).

The graphic geometric mean time curves for plasma concentration for Eudorlin^® ^extra compared to the reference products – Nurofen^® ^forte and Migränin^® ^were similar with a slightly higher and earlier geometric mean maximum for Eudorlin^® ^extra (see Figures 2 and 3).

### Safety

14 subjects of the safety population (N = 60) reported a total of 20 adverse events during the study, 6 of these subjects after treatment with Eudorlin^® ^extra, 6 after Nurofen^® ^forte and 4 after Migränin^®^. The only adverse event with a possible relation to the study drug was headache reported by 1 subject after intake of Migränin^®^. 19 were unrelated and 2 unlikely drug related.

## Discussion

The results of this study demonstrate that a single dose of Eudorlin^® ^extra results in similar total systemic exposure (as measured by AUC) compared to Nurofen^® ^forte and after Migränin^® ^throughout a 24 h period. However, the peak plasma concentration (C_max_) was higher and the time to peak plasma concentration (t_max_) shorter in comparison to the two reference formulations suggesting a more rapid absorption of the test formulation (for illustration see Figures 2 and 3). These results are consistent with the results of the dissolution testing which suggested a faster release of ibuprofen from the test formulation. All measures are however within the acceptance range of the EMEA bioequivalence guidance [3]. This asks to demonstrate that the 90% confidence interval of AUC_0-t(last)_, AUC_0-∞ _and C_max _lie within an acceptance range of 80 – 125% while t_max _should lie within a clinically determined range but is not further specified.

### Acceptance range peak plasma concentration (C_max_)

Ibuprofen has shown to have a low acute and chronic toxicity in a number of clinical studies and registries [2], in particular using the low dose tested. An evaluation of reported side effects after single doses in 15 double-blind randomized trials [4] demonstrated that these were equally distributed across all doses tested. Out of 707 patients 13 (1.8%) reported side effects, 4 of which reported GI-related adverse events. The study protocol considered bioequivalence to be acceptable when the 90% confidence interval was between 75 – 133% based on a large body of bioequivalence studies comparing generic formulations of 400 mg ibuprofen with the reference formulation (Brufen 400 mg in most cases) [5]. Additional file 1 displays the results from these studies, illustrating that the C_max _of generic formulations varies substantially from 28.7 (Motrin^® ^to 43.3 (Ibuprofen 400^® ^Stada).

C_max _values reported for the present study proved to be within the range of the wide (75 – 133%) but as well within the narrow confidence interval (80 – 125%) and are compatible with the other studies conducted [5], including the data of a recently published report by Bienert et al. [6].

### Rate and extent of absorption (C_max _and t_max_)

A quick release of ibuprofen in the gastrointestinal tract following oral administration is desirable [7] to achieve rapid pain relief and to avoid overdosing due to multiple ingestions based on a prolonged onset of action. As serum concentrations of ibuprofen and its analgesic effect are highly correlated, rapid ibuprofen absorption is the sole prerequisite for the quick onset of its action. Because its membrane permeability approaches up to 100% dissolution of the tablet becomes the rate limiting step for absorption [8]. A more rapid pain relief could translate in higher patient satisfaction and less danger of repeated ibuprofen intake due to slow onset of action.

It has been shown in the present study that Eudorlin^® ^extra tablets are rapidly dissolved in vitro resulting in 99% dissolution after only 5 minutes. In comparison it took 30 and 45 min respectively for the two reference formulations to achieve a similar degree of dissolution. This observation is compatible with the faster (t_max_) and higher peak plasma concentration (C_max_) observed in the present cross-over study in healthy volunteers. The values obtained did on the other hand not trespass the predefined acceptance range for C_max _as outlines above, thus not questioning the assumption of bioequivalence.

## Conclusion

Statistical analyses of primary parameters provided evidence for the therapeutic equivalence of the three ibuprofen formulations: the 90%-confidence intervals for the comparison Eudorlin^® ^extra/Nurofen^® ^forte and Eudorlin^® ^extra/Migränin^® ^were within the predefined acceptance range of 0.80 – 1.25 for both, AUC_0-t(last) _and AUC_0-∞ _and within the acceptance range of 0.75 – 1.33 for the parameter C_max_. Peak plasma concentration was however higher and the time to peak plasma concentration shorter, compatible with a more rapid pain relief with Eudorlin^® ^extra. All three formulations were well tolerated. The only adverse event with a possible relation to the study drug was reported by one subject after Migränin^® ^exposure.

## Methods

### Study design and inclusion criteria

This phase I study (study code 398B6) was a single-dose, randomized, open label, three-treatment, three-period, three-sequence cross-over with a wash-out period of 7–10 days. Patients were eligible for inclusion if they were male or female of Caucasian origin, between 18 and 55 years of age, a body mass index (BMI) of between 19 and 28 kg/m^2 ^and had provided written informed consent prior to study participation. Women of childbearing age had to be negative on a pregnancy test and had to agree to be either sexually inactive for 30 days prior to study participation or use an appropriate method of contraception.

### Test product

Film coated tablets of ibuprofen (Eudorlin^® ^extra for oral administration, batch number 62020, expiry date 05/2009) were tested against two reference formulations of ibuprofen: Nurofen^® ^forte (Ibuprofen 400 mg coated tablets, batch number 13J, expiry date 06/2009 and Migränin^® ^(Ibuprofen 400 mg coated tablets, batch number 12J, expiry date 06/2008). Dissolution testing was performed at pharm-analyt Labor GmbH, Baden, Austria between February 23^rd ^and March 15^th ^2007 using a HPLC-UV technique.

### Treatment protocol

**Figure 4:**
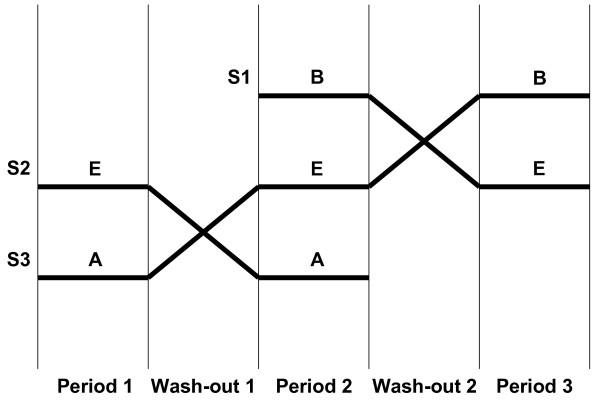
**Allocation of subjects to the appropriate sequence groups during the study**. S: Sequence groups; A: subjects receiving drug A (Nurofen^® ^forte), B: subjects receiving drug B (Migränin^®^, E: subjects receiving Eudorlin^® ^extra.

A three sequence cross-over design, allowed for the comparison of different subject sequences: 1) Subjects who received Nurofen^® ^forte (A) during periods 1 and 2 compared to subjects who received Eudorlin^® ^extra (E) during these periods (1 and 2) (sequence AE/EA); 2) Subjects who received Migränin^® ^(B) during period 2 and 3 compared to subjects who received Eudorlin^® ^extra (E) during these periods (2 and 3) (sequence EB/BE). The study consisted of three treatment phases separated by wash-out periods of 7 to 10 days (Figure 4). At least 12 h before each treatment period the subjects were admitted to the site. After an overnight fasting of at least 10 h subjects were administered 400 mg ibuprofen in sitting position between 8 and 9 a.m. (time point 0). Blood samples (6 ml each) were collected from all subjects 12 min prior to dosing and at 0.25, 0.5, 0.75, 1.0, 1.25, 1.5, 1.75, 2.0, 2.5, 3.0, 3.5, 4.0, 5.0, 6.0, 8.0, 10.0, 12.0, 16.0 and 24.0 hours after drug administration. Patients were observed for a total of 36 h (12 h prior until 24 h after drug administration). The blood samples were collected in heparinised tubes and cooled immediately in an ice bath and centrifuged at 3500 r.p.m. under refrigeration to obtain plasma. Immediately after centrifugation the plasma was homogenized, the tubes capped and stored frozen at -20°C.

### Clinical examination

Personal data, medical history, physical examinations (height, weight, 12-lead ECG), vital signs (blood pressure, heart rate, temperature) and laboratory tests were obtained during the screening phase. During each treatment period, vital signs and adverse events were recorded. Clinical safety parameters included a general medical examination, heart rate and blood pressure (sitting position) and the documentation of adverse events. Clinical laboratory safety was assessed with clinical chemistry, haematology, urinalysis, pregnancy test, HIV, HbsAg and HCV screening, urine drug screening and an alcohol breath test. Within a 7 day time frame after last blood sampling an ECG, clinical laboratory parameters and physical examination were repeated.

### Analytical method and chromatographic system

Determination was conducted considering GLP-guidelines [9]. Procedure of validation and acceptance criteria were based on the rules and guidelines of the FDA [10] and of the ICH Consensus Guidelines. Pre-study validation of the method was performed by documentation of specificity, linearity, limit of quantification, precision and accuracy. Furthermore recovery rate and stability of ibuprofen in plasma samples were investigated. For in-study validation each run contained samples for the calculation of standard curves; quality control samples and the samples of the subjects. The run was accepted, if no more than two of six quality control samples were outside ± 15% from the nominal value.

Determination of ibuprofen was performed using HPLC method with fluorescence detection [11]. A Merck-Hitachi L-7485 was used for detection of ibuprofen (excitation 225 nm, emission 290 nm). Chromatographic separation was carries out using a Luna 5 μC18(2) 100A, 50 × 2.0 mm (Phenomenex, USA). The mobile phase was 35% 0.02 molar phosphoric acid: 65% MeOH (v/v). The flow rate was 1.0 ml/min.

A sample volume of 250 μl was analyzed and a volume of 10 μl injected onto the chromatographic column. The method was validated between 0.200 and 75.0 μg/ml. In this study the retention time was 1.5 min. The blank sample showed no peaks in the retention time window of interest.

The assay showed an acceptable *linearity *over a concentration range from 0.200 to 75.0 μg/ml (r ≥ 0.99, n = 8). The limit of detection defined as baseline noise was 0.206 μg/l. *Specificity: *There were no interfering peaks for the endogenous compound for the blank plasma at the retention time of ibuprofen (1.5 min.). The inter-batch *precision *was between 9.96% for 0.500 μg/ml and 6.35% for 63.8 μg/ml. The within-batch precision was between 1.56% for 0.206 μg/ml and 5.83% for 75.0 μg/ml. The *accuracy *(bias) for ibuprofen was expressed as a percent deviation of observed plasma concentration from theoretical concentration (0.5, 5.51 and 63.8 μg/ml). The accuracy ranged from 1.3% (0.500 μg/ml) to 4.5% (5.51 μg/ml). For the determination of *recovery *peak areas determined following analysis of calibration standards with concentrations of 0.5, 5.51 and 63.8 μg/ml were compared with data obtained by direct injection of aqueous solutions of these concentrations. The mean recovery of ibuprofen from spiked plasma samples was on average 92.2%.

### Statistics

Sample size was based on a intra-individual CV of 26% based on a previous bioavailability study of Eudorlin^® ^extra (Clinical trial report BCBe/02/Ibu-BV-001) and data from Blume and Mutschler [5]. For a total of 36 subjects (2 × 18) the power to detect bioequivalence was 96% assuming a true ratio of 0.95, and 98% assuming a true ratio of 1.05 (calculations based on equivalence limits of 0.75 and 1.33). As bioequivalence had to be shown with 2 reference products sample size was adjusted to 3 × 18 (54) subjects. Therefore 60 patients were included to allow a drop-out of 10% (6 subjects).

The statistical analyses were performed by using SAS 8.2 [12]. The primary pharmacokinetic parameters after logarithmic transformation (multiplicative model) were subjected to an analysis of variance (ANOVA) with the factors SEQUENCE, SUBJECT nested within SEQUENCE, PERIOD and FORM (drug formulation) using a general linear model procedure. For assessment of bioequivalence 90%-confidence intervals for the formulation ratio in the parameters AUC_t _(calculated using the trapezoidal rule), AUC_∞ _(calculated as sum of AUC_t _and the extrapolated area using the last measured concentration [C_(last)_] and the elimination half life by taking the formula: [C_(last) _× t_1/2_/ln2]), and C_max _(directly obtained from measured values) of ibuprofen were calculated using the ln-transformed data. Bioequivalence was accepted if the calculated 90%-confidence intervals were within 0.80 – 1.25 for AUC_t _and within 0.75 – 1.33 for C_max_. Possible side effects of the study medication and any adverse events were listed. Time of maximum plasma concentration (t_max_) was directly obtained from measured values and compared using the two samples Wilcoxon test. Elimination half life (t_1/2_) was calculated from concentrations of the elimination phase using semi-log transformed data and linear regression was documented individually.

## Authors' contributions

AG was the principal investigator at IFE Human Pharmacology SRL and was responsible for planning and conducting the study. PB drafted the manuscript. All authors read and approved the final manuscript.

## Supplementary Material

Additional file 1 **Cmax values from various bioequivalence studies** Click here for file

## References

[B1] The story of ibuprofen. http://www.ibuprofen-foundation.com/.

[B2] Frölich JC, Fricker RM, Frölich JC, Kirch W (2006). [Pain therapy and analgetics-antipyretics (Nonsteroidal antirheumatic drugs – NSAR)]. Praktische Arzneitherapie.

[B3] Note for Guidance on the Investigation Bioavailability and Bioequivalence (CPMP/EWP/QWP/1401/98).

[B4] Furey SA, Waksman JA, Dash BH (1992). Nonprescription ibuprofen: side effect profile. Pharmacotherapy.

[B5] Blume H, Mutschler E (1996). Bioäquivalenz, Qualitätsbewertung wirkstoffgleicher Fertigarzneimittel.

[B6] Bienert A, Szkutnik-Fiedler D, Dyderski S, Grzeskowiak E, Drobnik L, Wolc A, Slawiniska U (2006). Comparative bioavailability study of two ibuprofen preparations after oral administration in healthy volunteers. Arzneimittel-Forschung.

[B7] Laska EM, Sunshine A, Marrero I, Olson N, Siegel C, McCormick N (1986). The correlation between blood levels of ibuprofen and clinical analgesic response. Clin Pharmacol Ther.

[B8] Newa M, Bhandari KH, Kim JO, Im JS, Kim JA, Yoo BK, Woo JS, Choi HG, Yong CS (2008). Enhancement of solubility, dissolution and bioavailability of ibuprofen in solid dispersion systems. Chem Pharm Bull (Tokyo).

[B9] GLP Principles of Good Laboratory Practice as specified by national (German Chemicals Law, Annex 1, 20 June 2002) and international (OECD, Paris, 1998; EC Directive 2004/10/EC, 11. February 2004).

[B10] U.S. Department of Health and Human Services Food and Drug Administration, Center for Drug Evaluation and Research (CDER), Center for Veterinary Medicine (CVM). Guidance for Industry Bioanalytical Method Validation. http://www.fda.gov/cder/guidance/index.htm.

[B11] Sochor J, Klimes J, Sedlacek J, Zahradnicek M (1995). Determination of ibuprofen in erythrocytes and plasma by high performance liquid chromatography. J Pharm Biomed Anal.

[B12] SAS8.2 (2001). SAS^® ^software. Release 82 edition.

