# Supplementary material for: Bioequivalence study of three ibuprofen formulations after single dose administration in healthy volunteers
Source: BMC Pharmacol. 2008 Oct 29;8:18. doi: 10.1186/1471-2210-8-18 (PMC2613135; doi:10.1186/1471-2210-8-18)
Supplement: Additional file 1 — Cmax values from various bioequivalence studies [file 1471-2210-8-18-S1.doc]

### Additional file 1 – C_max_ values from various bioequivalence studies

9 bioequivalence studies reporting C_max_ values for the release of Ibuprofen from tablets (modified from [7])

| **Study** | **Product** | **C_max_** | **CV (%)** | **N** |
| --- | --- | --- | --- | --- |
| 1. | Dolgit® | 40.6 | 24 | 8 |
|  | Brufen® 400 | 39.4 | 25 | 8 |
| 2. | Ibol® 400 | 37.6 | 20 | 12 |
|  | Brufen® 400 | 40.7 | 22 | 12 |
| 3. | Parsal® 400 | 30.5 | 20 | 18 |
|  | Brufen® 400 | 34.3 | 34 | 18 |
| 4. | Dualbuprofen® 400 | 23.8 | 21 | 18 |
|  | Motrin® | 28.7 | 21 | 18 |
| 5. | Ibuphlogont® 400 | 42.7 | 30 | 24 |
|  | Ibu-Attritin® | 38.1 | 26 | 24 |
| 6. | Ibuprofen 400® Stada | 43.3 | 28 | 16 |
|  | Ibuprofen Klinge 400® | 33.6 | 28 | 16 |
| 7. | Ibuprof® 400 | 31.9 | 26 | 12 |
|  | Dolgit® 400 | 38.2 | 19 | 12 |
| 8. | Tabalon® 400 | 38.6 | 15 | 10 |
|  | Brufen® 400 | 43.1 | 17 | 10 |
| 9. | Urem® forte | 38.5 | 20 | 12 |
|  | Brufen® 400 | 39.9 | 23 | 12 |

| 10. (present study) | Eudorlin® extra | 36.6 | 17 | 60* |
| --- | --- | --- | --- | --- |
|  | Nurofen® forte | 32.9 | 18 | 40 |
| 11. (present study) | Eudorlin® extra | 35.9 | 18 | 60* |
|  | Migränin® | 30.9 | 20 | 40 |

* overall 60 patients in this study received Eudorlin® extra, 40 received Nurofen® forte and 40 received Migränin®
